# The LRP4/YAP axis drives the radiation-tolerant persister (RTP) cell state in breast cancer

**DOI:** 10.7150/thno.101393

**Published:** 2025-06-23

**Authors:** Violaine Forissier, Julien Wicinski, Martin Castagné, Guillaume Pinna, Shuheng Lin, Anaïs Grandon, Caroline Bonnet, Manon Macario, Remy Castellano, Simon Valdenaire, Julien Darréon, Agnès Tallet, Christophe Ginestier, Emmanuelle Charafe-Jauffret

**Affiliations:** 1 CRCM, Inserm, CNRS, Institut Paoli-Calmettes, Aix-Marseille Univ, Epithelial Stem Cells an Cancer Lab, Equipe labellisée LIGUE contre le cancer, Marseille, France.; 2 Plateforme ARN Interférence (PARI), Université Paris Cité, Inserm, CEA Stabilité Génétique Cellules Souches et Radiations, Fontenay-aux-Roses, France.; 3 Aix-Marseille Univ, Inserm, CNRS, Institut Paoli-Calmettes, TrGET Plateform, Marseille, France.; 4 Department of Medical Physics, Institut Paoli-Calmettes, Marseille, France.; 5 Department of Oncology Radiation Therapy, Institut Paoli-Calmettes, Marseille, France

**Keywords:** breast cancer, cancer stem cell, radiation therapy, LRP4, YAP, cell plasticity

## Abstract

**Rationale:** Breast cancer recurrences and treatment failures can be attributed to intra-tumoral heterogeneity (ITH), which is characterize by the coexistence of diverse cellular states, including cancer stem cells (CSCs), within a single tumor.Recent insights suggest that ITH arises from non-genetic dynamics, enabling tumors to adapt and evolve into a therapy-tolerant state under treatment pressure. The aim of this work is to decipher the origin of persistent radiation tolerant cells (RTP) in breast tumors and to understand their mechanisms in order to find new strategies to avoid radiation resistance.

**Methods:** To this aim,we developed a lineage tracing system and engineeredvarious breast cancer cell lines and patient-derived xenografts totracked radiation-induced cell plasticity. We combined lineage tracing with a unique RNAi screen under irradiation to identify and functionally validate the regulators of radio-induced cell plasticity.

**Results:** We discovered that RTP cells, which possess CSC properties, emerge from radiotherapy-induced reprogramming of non-CSCs. From the combinatorial approach of the lineage tracing and the RNAi screen under irradiation, we then identified and functionally validated the LRP4/YAP axis as a crucial regulator of radio-induced cell plasticity. We further demonstrate that overexpression of LRP4 is common in residual disease post-treatment and is associated with breast tumors of poor prognosis.

**Conclusions:** This work has demonstrated that the LRP4/YAP axis drives radioresistance by promoting the emergence of RTP cells through radiation-induced plasticity, and that modulation of the LRP4/YAP axis is a promising strategy for sensitizing breast cancers to radiotherapy, opening up a new avenue for improving patient outcomes.

## Introduction

Breast cancer is the most common cancer worldwide 1 and a public health challenge in many countries. Over the past decade, improvement in therapeutic care has dramatically change the treatment landscape of breast cancer patients. Thanks to these advances, in a growing number of cases, breast cancers previously known as poor prognosis can now be considered as a “chronic” pathology, with successive periods of therapeutic response and recurrence. Nevertheless, this chronicization of the disease merely delays the emergence of an incurable life-threatening disease with a progressive decrease in patient overall survival after no, 1, or 2 recurrences 2. The mechanisms behind this failure are multiple, and may explain our difficulties in treating relapsing patients. Intratumor heterogeneity (ITH) is one of the most recent avenues explored to explain the origin of recurrence, the tumor being seen as a complex ecosystem composed of different tumor states more or less likely to respond to therapy, all evolving dynamically under the pressure of treatment 3. The cancer stem cell (CSC) state is one such tumor state, capable of fueling tumor growth and resistance to therapy, making tumor relapse possible 4. A more complex degree of ITH exists beyond the concept of CSCs, resulting from non-genetic dynamics of cellular states, spontaneous or induced by therapy, supporting the hypothesis that cancer cells could evolve phenotypically towards a therapy-tolerant state, potentially offering a survival advantage against treatment 5. With radiotherapy (RT) considered as a standard of local care with over 85% of breast cancer patients receiving RT 6, it is crucial to evaluate the effect of RT on cellular plasticity that leads to tumor adaptation producing cells with molecular programs that contribute to treatment failure and recurrence. In addition, RT is also a standard of palliative care in specific metastatic locations such as the spinal cord, and is becoming of paramount importance for the ablative treatment of oligo-metastases, with a gain in survival, reinforcing the need to understand how to radiosensitize a complex tumor ecosystem 7-10.

If seminal works have identified an enrichment in CSC in response to RT 11,12, the origin of this radiation-tolerant persister cells remain elusive. Previous work has suggested that RT induces reprogramming of breast cancer cell through epithelial-mesenchymal transition 13 and that radiation-tolerant CSCs could also arise from the transformation of more mature cancer cells 14 potentially explaining the emergence of recurrences post-irradiation despite an objective therapeutic response. However, very little is known about the rules that determine how a heterogeneous population reacts to radiation, and the dynamics of transition between radiosensitive and radiotolerant state. Disrupting these radio-induced cellular state transitions could be a promising strategy for avoiding difficult-to-manage recurrences in breast cancer treatment.

In this work, we set up a lineage tracing system to monitor radio-induced cell plasticity in different breast cancer models. We showed that radiotolerant persister cells originate from non-CSC reprogramming. Using an original RNAi screen setting based on lineage tracing under irradiation, we identified and functionally validated the LRP4/YAP axis as key regulator of the radio-induced cell plasticity, paving the way for therapeutic perspectives in cancer treatment.

## Results

### Radiation therapy induced a transient increase of the ALDH^br^ breast cancer stem cell population

**Figure 1:**
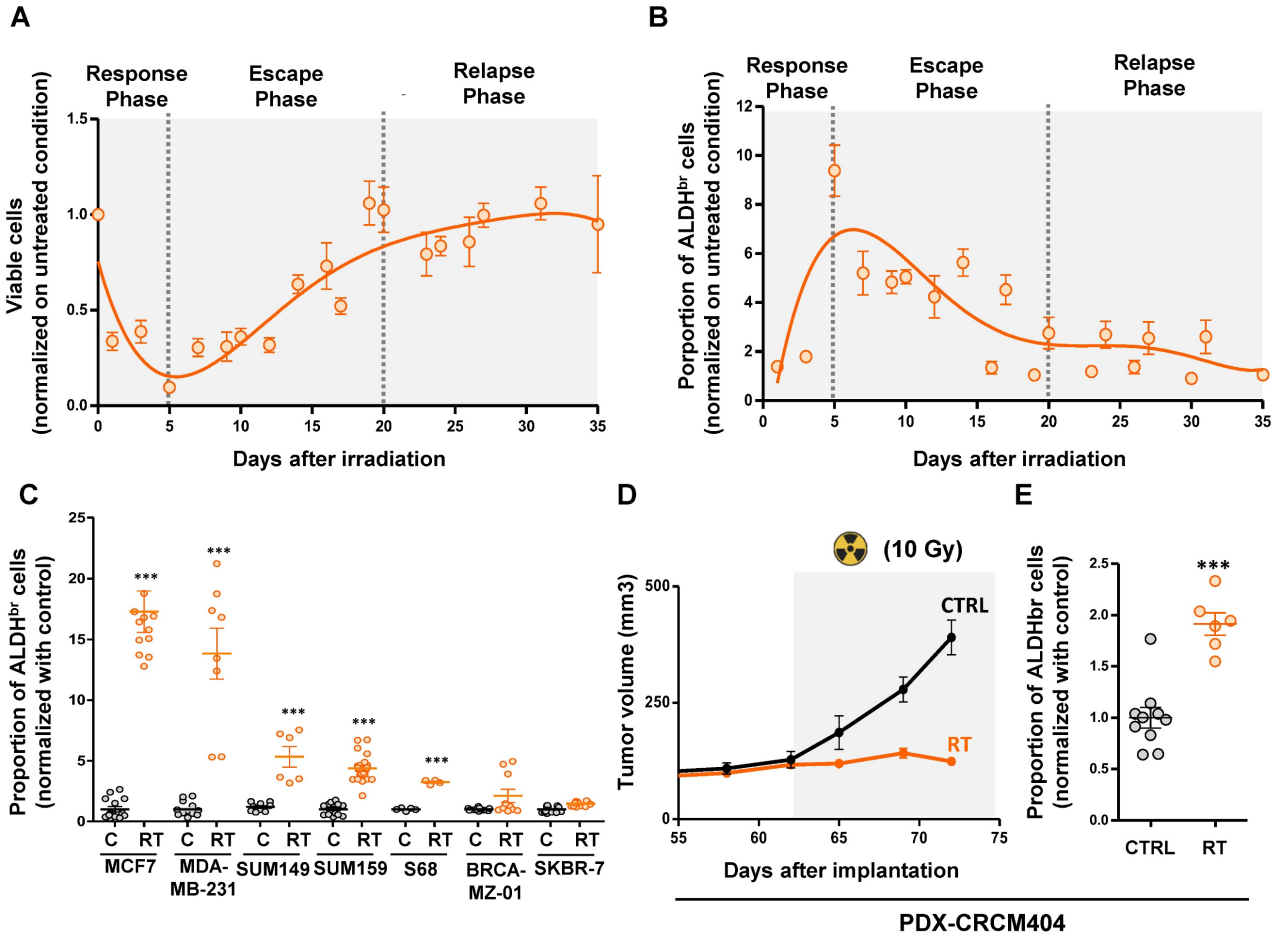
** Radiation-tolerant persister cells are enriched in ALDH^br^ cells. A-B.** Cinetic curves tracing the proportion of viable cells (**A**) or ALDH^br^ cells (**B**) following irradiation (10Gy), in SUM159 cells. **C**. Proportion of ALDH^br^ cells in untreated condition (C) compare to irradiated cells (RT) in seven different breast cancer cell lines. **D**. Effect of radiation therapy (RT) on the tumor growth of PDX-CRCM404, compared to the untreated condition (C). The gray area corresponds to the post-treatment period. **E.** Proportion of ALDH^br^ cells in untreated condition (C) compare to irradiated cells (RT) in PDX-CRCM404, 10 days post-irradiation. Statistical test used is Student's t-test. Data represent mean ± SD. *p<0.05, **p<0.01, ***p<0.001.

In order to evaluate the kinetics of breast cancer stem cell enrichment after radiation therapy, we monitored the total cell viability and the ALDH^br^ cell proportion in SUM159 breast cancer cells during 35 days post-irradiation. Of note, SUM159 cells harboring a high ALDH enzymatic activity (ALDH^br^) have been functionally demonstrated to be enriched in CSC compared to the cell population presenting a low ALDH activity (ALDH^-^) 15. We exposed cells to 10Gy using a MeV electron beam of an Elekta Synergy linear accelerator, approximating settings use in clinics to treat breast cancer patients. Under these conditions, the total cell population evolved in three phases. A first response phase showed a dramatic decrease of the cell viability (day 0 to day 5) reaching below 10% of residual cells. That phase was followed by an escape phase (day 5 to day 20), where residual cells restart to grow until reaching the initial number of viable cells before irradiation. Then, we observed a relapse phase (day 20 to day 35) were cells continue to grow with the same rate observed in the non-irradiated condition (**Figure 1A**).

As expected, we observed during the response phase a significant increase of the breast CSC proportion with 9.2-fold more ALDH^br^ cells at day 5 compared to untreated condition (**Figure 1B**). Surprisingly, while the total number of cells increases again during the escape and relapse phases, the proportion of ALDH^br^ cells declines to reach the initial steady state observed in the non-irradiated condition, demonstrating only a transient increase in breast CSCs post-irradiation. To extend our observation, we monitored the proportion of ALDH^br^ cells within a panel of seven different breast cancer cell lines representing the molecular diversity of breast cancers. In 5 out 7 cell line models tested we did observe a significant increase of the ALDH^br^ cell population 5 days post-irradiation ranging from a 3.8-fold to a 17.2-fold increase compared to untreated conditions (**Figure 1 C**).

We next evaluate the proportion of ALDH^br^ cell under radiation therapy in a patient-derived xenograft (PDX-CRCM404) based on a protocol of CT scanning and radiation dose optimization to be as close as possible to clinical practice (**Supplementary Figure 1**). PDX-CRCM404 cells xenografted into the mammary fat-pad of NOD/SCID mice were irradiated as soon as each tumor reaches a volume of 300 mm^3^ and the proportion of ALDH^br^ cell was measured 10 days post-irradiation. Compared with untreated conditions, irradiation of PDX-CRCM404 induces tumor flattening (**Figure 1D**) which is accompanied with a significant increase of the ALDH^br^ cell population (**Figure 1E**). These observations confirm *in vivo* that irradiation increases the proportion of ALDH^br^ cells while tumor growth is controlled by treatment.

Altogether, our observations indicate that radiation-tolerant persister cells are highly enriched in ALDH^br^ cells, further questioning the cellular and molecular mechanisms sustaining the emergence of this cell population.

### ALDH^br^ radiation-tolerant persister cells originate from ALDH^-^non-CSC compartment

**Figure 2:**
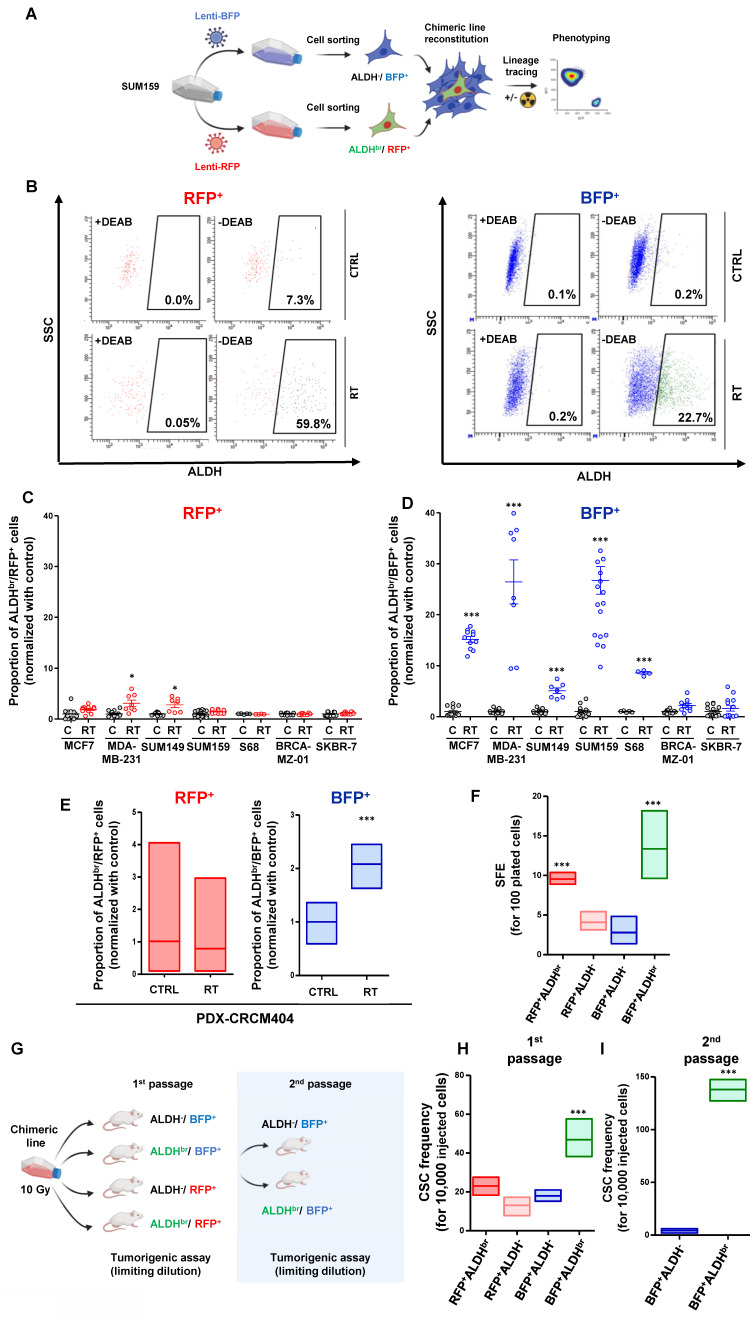
** Radiation therapy induces a cell state transition with the generation of induced cancer stem cells (iCSC). A.** Schematic representation of the lineage tracing system protocol. **B.** Representative examples of FACS plot for ALDH activity in SUM159 engineered with lineage tracing system in untreated (CTRL) and irradiated conditions (RT), within RFP^+^ or BFP^+^ subpopulation. DEAB is an ALDH inhibitor used as negative control. **C-D.** Proportion of ALDH^br^ cells in untreated condition (C) compare to irradiated cells (RT), in the RFP^+^ (**C**) and the BFP^+^ (**D**) cell population from seven breast cancer cell line engineered with the lineage tracing system. Statistical test used is Student's t-test. Data represent mean ± SD. **E.** Proportion of ALDH^br^ cells in PDX-CRCM404 engineered with the lineage tracing system in untreated (CTRL) and irradiated (RT) conditions, in the RFP^+^ and the BFP^+^ cell subpopulations. Statistical test used is Student's t-test. Data represent mean ± IC.**F.** Tumorsphere-forming efficiency (SFE) of each FACS-sorted SUM159 cell subpopulation following irradiation. Data represent mean ± IC. Statistical test used is pairwise chi-square test.** G**. Schematic representation of the in vivo experimental design. **H-I**. Box plots display CSC frequency calculated using an extreme limiting dilution analysis (ELDA) after one (**H**) and two passages (**I**). Results are expressed as the estimated number of CSCs for 10,000 tumor cells. Data represent mean ± IC. Statistical test used is pairwise chi-square test. *p<0.05, **p<0.01, ***p<0.001.

In order to identify the cellular origins of the ALDH^br^ cell population post-irradiation, we developed a lineage tracing system to engineer breast cancer cell lines (**Figure 2A**). With this system, we can follow the progeny of CSCs (ALDH^br^/RFP^+^) and non-CSCs (ALDH^-^/BFP^+^) under irradiation using flow cytometry (**Figure 2B, Supplementary Figure 2A**). We engineered a total of seven breast cancer cell lines that have been exposed to radiation. Five days post-irradiation, the proportion of ALDH^br^/RFP^+^ cells (the so-called “native CSC”) were stable in five cell lines and slightly increased in MDA-MB-231 and SUM149 compared to the untreated conditions (**Figure 2C**). In contrast, a significant increase in ALDH^br^/BFP^+^ cells was identified in most cell lines post-irradiation, supporting the emergence of ALDH^br^ cells observed under irradiation (**Figure 2D**). Using similar approach, we engineered PDX-CRCM404 and confirmed that the increase in ALDH^br^ cells was due to the phenotypic conversion of cells initially ALDH^-^ (**Figure 2E**). Thus, the emergence of ALDH^br^ radiation-tolerant persister cells is driven by a phenotypic conversion of ALDH^-^ cells rather than an innate resistance of native CSC to radiation.

To address whether this phenotypic conversion is translated into a functional change, we assessed the tumorsphere-forming capacity of each SUM159 cell subpopulation post-irradiation. As expected, native CSCs (ALDH^br^ /RFP^+^) presented an increase in tumorsphere-forming capacity compared to non-CSCs (ALDH^-^/BFP^+^) and the ALDH^-^ progenies (ALDH^-^/RFP^+^) generated from the differentiation of native CSCs. Interestingly, the ALDH^br^ cells generated from the phenotypic conversion of the non-CSC (ALDH^br^ /BFP^+^) presented the higher capacity to form tumorsphere with 14 tumorspheres formed for 100 cells plated (**Figure 2F**). These results suggest that this phenotypic conversion reflect a functional shift yielding cells with CSC properties. To further validate this observation, we performed a limiting dilution transplantation assay into the fat pad of NOD/SCID mice for each FACS-sorted SUM159 cell subpopulation, 5 days after irradiation (**Figure 2G**). Tumorigenicity is directly related to the presence of CSCs, and this assay gives an estimate of the proportion of residual tumorigenic CSCs 16. The ALDH^br^ /BFP^+^ cells presented the highest tumorigenic potential with an estimated breast CSC frequency of 42 CSC out of 10,000 cells compared to 2-fold less in all the other cell subpopulations (**Figure 2H, Supplementary Figure 2B**). This observation was further exacerbated after a transplantation into secondary mice, demonstrating the self-renewal and differentiation potential of this cell subpopulation (**Figure 2I**).

Altogether, we showed that the increase in ALDH^br^ cells after irradiation was not due to an intrinsic resistance of native CSCs but rather to a radio-induced cell state transition, yielding reprogrammed cells with CSC properties. These induced-CSCs (hereafter named iCSC) could potentially participate to tumor recurrences after treatment.

### iCSC harbored a unique cell state identity

**Figure 3:**
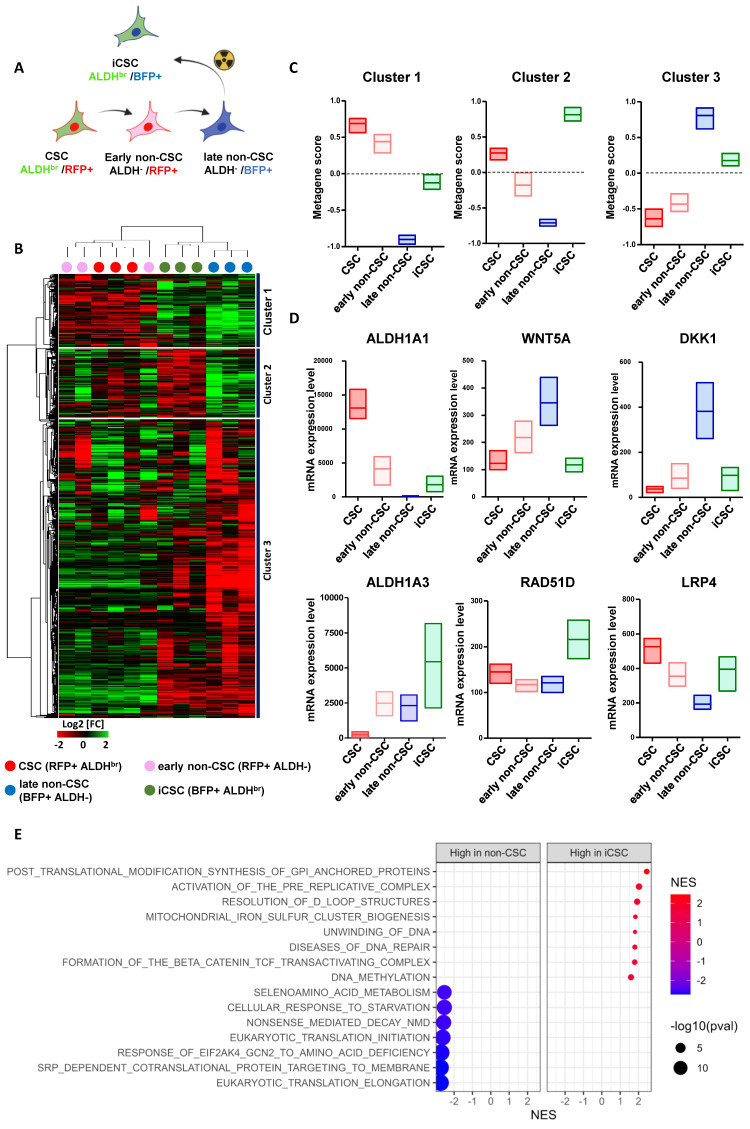
** Gene expression profiling of each cancer cell state after irradiation. A.** Schematic representation of the subpopulation transition dynamics and predictive contribution of each population under radiation therapy or basal state. **B.** Hierarchical clustering of each SUM159 cell subpopulation FACS-sorted five days after irradiation, based on mRNA expression levels. The dendrogram of samples represents overall similarities in gene expression profiles. Three large groups of samples are evidenced by clustering. Cell subpopulation are color-coded as follows: red for CSC, pink for early non-CSC, blue for late non-CSC, and green for iCSC. The dendrogram of mRNA expression level represent genes association with three main clusters of genes coexpressed. **C**. Box plots representing the gene-expression level of each cluster metagenes in each SUM159 cell subpopulations post-irradiation. **D**. Box plots representing the gene-expression level of six selected genes in each SUM159 cell subpopulations post-irradiation. **E**. Bubble graph for GSEA-based Reactome analysis revealed significantly enriched pathways in late non-CSC compared to iCSC.

We hypothesized that the emergence of iCSC arises from transcriptional reactivation of native CSC-linked genes. We thus assessed the transcriptional status of each SUM159 cell subpopulations. To that end, we established cell state gene expression profiles using RNA-sequencing of each four different FACS-sorted subpopulations post-irradiation: the native CSC (ALDH^br^ /RFP^+^), the two non-CSCs subpopulations issued either from the native CSC differentiation (ALDH^-^/RFP^+,^ hereafter named early non-CSC) or from native non-CSC progeny (ALDH^-^/BFP^+,^ hereafter named late non-CSC), and the iCSC issued from native non-CSC conversion (ALDHbr /BFP^+^) (**Figure 3A**). Gene expression clustering revealed three main groups of co-expressed genes (cluster 1, 2, and 3) that define each cell states (**Figure 3B, see supplementary Table 1**).

We performed a metagene analysis for the global expression of genes defining each cluster. Using the metagene score, we ranked each cell subpopulation and observed a significant association and high expression of metagene generated from cluster 1 in native CSC that is progressively downregulated in early non-CSC before being totally silent in late non-CSC (**Figure 3C**). Conversely, metagene originating from cluster 3 display an opposite trend, with a high expression in late non-CSCs and a downregulation in native CSCs (**Figure 3E**). These results support the progressive differentiation of the native CSC into late non-CSC observed in culture using our lineage tracing system. Interestingly, iCSCs appear to downregulate the metagene derived from cluster 3, which defines the molecular identity of their former cell state. Furthermore, iCSCs seem to partially express the metagene originating from cluster 1, which is associated with the CSC state, and overexpress the metagene from cluster 2, which appears to be a specific marker for this iCSC state (**Figure 3D**). These observations support the idea that during irradiated non-CSCs reprograming, rather than a cellular process restoring a stemness program originally activated in native CSCs, iCSCs acquire a distinct transcriptional identity. Indeed, a GSEA analysis revealed that iCSC share some common stem cell program with native CSC while harboring the activation of specific pathways such as PI3K/AKt (**Supplementary Figure 3A**). This iCSC state is in part defined by an overexpression of ALDH1A3 (and to a less extent ALDH1A1) explaining the ALDH^br^ enzymatic activity observed in radiation-tolerant persister cells (**Figure 3E**). A GSEA analysis identified that iCSC compared to late non-CSC were significantly correlated with DNA repair-related genes (e.g. RAD51D) potentially explaining the increase capacity of these cells to resist radiotherapy (**Figure 3F**). We also observed an association with genes involved in the activity of the β-catenin/TCF complex known as effector transcription factors downstream of the WNT signaling pathways (e.g. DKK1, WNT5A, LRP4) (**Figure 3E**). Since drug-tolerant persister (DTP) cells exhibit a diapause-like state, we investigated whether radiation-tolerant persister cells couldbe enriched in a similar state 17. However, we could not confirm this hypothesis by evaluating the association of the iCSC signature with diapause-related genes identified in DTP (**Supplementary Figure 3B**).

These results indicate that iCSC is a specific cell state with its own transcriptional identity excluding a simple reacquisition of native cancer stemness program or the induction of a diapause-like state resembling the one activated in DTP.

### RNAi screen to identify effectors of radiation-induced cell state transition

**Figure 4:**
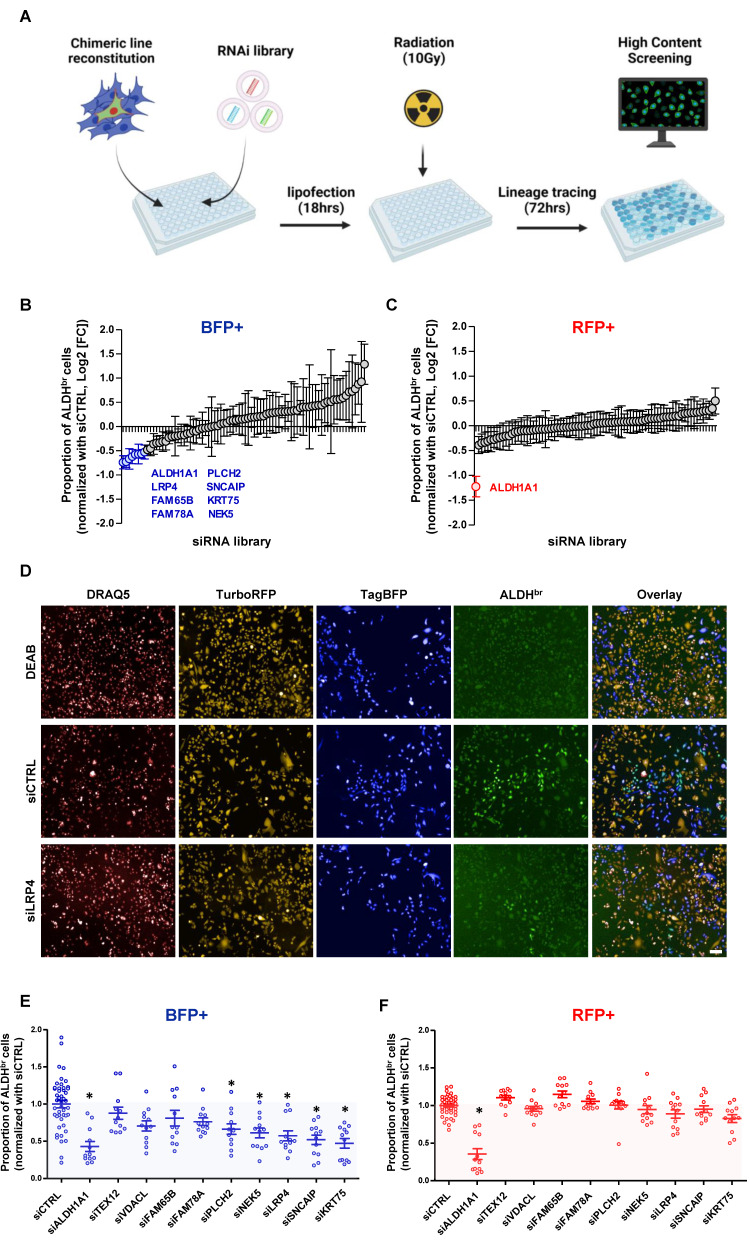
** A RNAi screen for the identification of functional regulator of radiation-induced cell plasticity. A**. schematic representation of RNAi screening strategy. **B-C**. Proportion of ALDH^br^ cells in BFP^+^ (**B**) and RFP^+^ SUM159 cells (**C**) following silencing of each individual gene contained in the RNAi library and normalized with non-targeting siRNA (siCTRL). Genes targeted by a siRNA inducing a significant reduction of the ALDH^br^ cell proportion are highlighted. Statistical test used is Student's t-test. Data represent mean ± SD** D**. Representative images of the high-content screening captures. ALDEFLUOR cellular staining is represented in green. Dead cells are labeled by DRAQ5 in red. RFP+ cells are in yellow and BFP+ cells in blue. **E-F**. Validation of candidate genes using FACS analysis. Proportion of ALDH^br^ cells in BFP^+^ (**E**) and RFP^+^ SUM159 cells (**F**) are represented normalized with non-targeting siRNA (siCTRL). siRNA targeting ALDH1A1 was used as positive control and siRNA targeting TEXT12 or VDACL were used as negative control. Statistical test used is Student's t-test. Data represent mean ± SD. *p<0.05, **p<0.01, ***p<0.001.

In order to functionally validate the regulators of radiation-induced cell state transition, we first defined the differentially expressed genes between late non-CSCs (ALDH^-^/BFP^+^) and the iCSCs (ALDH^br^ /BFP^+^) (**Supplementary Figure 4**). Then, we carried out an RNAi screen using a custom RNAi pool library targeting the top 80 overexpressed genes in iCSC compared to late non-CSC (4 pooled RNAi/gene), in the SUM159 cells engineered with the lineage tracing system. This approach adapted from previous works 18,19 allows the concurrent measurement of changes in cell state proportion following irradiation and upon gene knockdown (KD). Cell state phenotypes were measured by high content screening to evaluate for each cell (DRAQ5+) their respective expression for RFP, BFP, and ALDH enzymatic activity (**Figure 4A**). As a positive control, silencing of ALDH1A1 hindered the appearance of ALDH^br^ cells in RFP+ and BFP+ cell populations. In the BFP+ population, silencing of 9 out 80 selected genes (TEXT12, VDACL, LRP4, FAM65B, FAM78A, PLCH2, SNCAIP, KRT75, NEK5) impaired the emergence of iCSC, whereas none of these genes silencing excepted ALDH1A1impacted the native CSC proportion post-irradiation (RFP+ cells) (**Figure 4B, C**).

Next, we generated a focused siRNA library targeting all candidate genes selected from the primary screen, and we performed validation experiments using flow cytometry analysis. The effect of gene silencing on the abrogation of iCSC emergence was confirmed for 5 out of 9 candidates (PLCH2, NEK5, LRP4, SNCAIP, KRT75), with no effect on the ALDH^br^ /RFP^+^ native CSC population with the exception of ALDH1A1(**Figure 4E, F**).

Altogether, we functionally validated 5 candidate genes as regulators of the radiation-induced cell state transition offering new potential actionable targets to increase breast cancer response to radiotherapy.

### LRP4/YAP axis controls the emergence of iCSC

**Figure 5:**
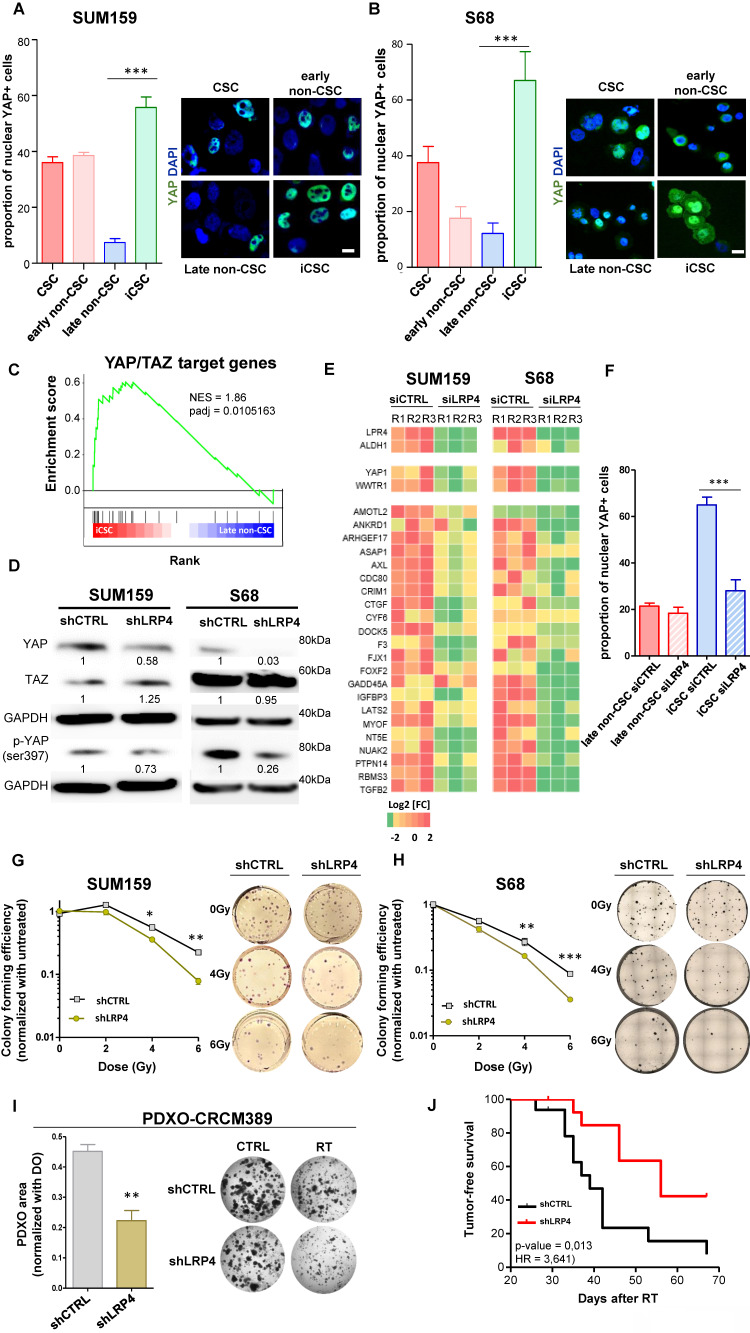
** LRP4/YAP axis inhibition radiosensitizes iCSC. A-B**. Proportion of cells (**A**, SUM159; **B**, S68) presenting a nuclear location of YAP in each cell subpopulations five days post-irradiation, with representative images of YAP staining (in green) in each cell subpopulations on the right panel. Nuclei are counterstained with DAPi (in blue).**C**. Pre-ranked GSEA interrogating differential expression between iCSC and late non-CSC and YAP/TAZ target genes. **D**. Western blot of markers related to YAP/TAZ signaling and its activation in SUM159 and S68 cells silenced for LRP4 (shLRP4) compared to the non-targeting shRNA (shCTRL). The mean intensities are indicated below each band for each condition. **E**. Heat map representing the mRNA expression of the LRP4, ALDH1A1, and YAP/TAZ target genes in SUM159 and S68 silenced for LRP4 (siLRP4) compared to the non-targeting siRNA (siCTRL). Each row represents three independent replicates per conditions (R1, R2, and R3). **F**. Proportion of SUM159 cells presenting a nuclear location of YAP in late non-CSC and iCSC following LRP4 silencing (siLRP4) compared to a non-targeting siRNA (siCTRL). **G-H**. SUM159 (**G**) and S68 cells (**H**), following LRP4 silencing (shLRP4) compared to a non-targeting siRNA (shCTRL), were exposed to various dose of radiation therapy and subjected to clonogenic survival assays (**G**), with representative images (right panels). **I**. Patient-derived xenograft organoid (PDXO) size distribution for CRCM389 cells WT (shCTRL) or silenced for LRP4 (shLRP4) following irradiation (RT) and compared to untreated condition (CTRL) (left panel). Representative pictures of PDXO-CRCM389 7 days post-treatment (right panel). Statistical test used is Student's t-test. Data represent mean ± SD. ns (not significant), *p<0.05, **p<0.01, ***p<0.001. **J**. Kaplan-Meier tumor-free survival curves of mice xenografted with 100,000-200,000 SUM159 irradiated cells silenced for LRP4 (shLRP4) compared to the control (shCTRL). p-value and hazard ration (HR) estimated according to Log-rank (Mantel-Cox) test.

Among the different candidate genes, we focused on LRP4 a modulator of Wnt/β-catenin signaling 20. We and others have already demonstrated the key role of WNT pathway in the regulation of breast CSC fate 19,21,22. Moreover, it was recently demonstrated that the activation of the transcription factor YAP through LRP4 confer tumorigenic potential to non-CSCs in pancreatic ductal adenocarcinoma 23. Furthermore, it has been widely demonstrated that YAP/TAZ can reprogram cancer cells into cancer stem cells and incite tumor initiation, progression and metastasis 24. Thus, we hypothesized that the LRP4/YAP axis may be a main actor of iCSC emergence under radiation therapy. Because YAP need to translocate to the nucleus to activate stemness program, we first tested YAP nuclear location in each cell subpopulations. We found a higher number of YAP+ nuclei in iCSCs compared with the late non-CSCs from which they were derived, suggesting that the emergence of radiation-tolerant persister cells is accompanied by an activation of YAP signaling (**Figure 5A, B**). Of note, native CSCs presented also a YAP activation, but to a lesser extent than iCSC, further confirming the central role of YAP pathway in regulating stemness. We performed a gene set enrichment analysis (GSEA) on RNA-seq data generated from iCSC and late non-CSC. We showed that iCSC cells were positively associated with YAP/TAZ target genes (NES = 1.86, p-adj = 0.0105163, **Figure 5C**). To evaluate the capacity of LRP4 to activate YAP, we knock-down LRP4 gene expression in SUM159 and S68 cells, using shRNA lentiviral constructs. We first assessed the protein expression of YAP/TAZ and the phosphorylation state of YAP. Compared to shCTRL, the knock-down of LRP4 significantly reduced YAP protein expression including its phosphorylated form (**Figure 5D**). Moreover, inhibition of LRP4 was enough to downregulate a core of 22 YAP/TAZ target genes 24 (**Figure 5E**). Furthermore, inhibition of LRP4 was enough to prevent the translocation of YAP in the nucleus of iCSC without affecting basal nuclear YAP observed in late non-CSC (**Figure 5F**).

To evaluate the functional consequences of LRP4/YAP axis inhibition on the response to radiation therapy, we evaluated the colony-formation efficiency post-irradiation of cells silenced for LRP4 expression compared to LRP4-expressing cells. We observed that LRP4 knockdown significantly impaired the ability of irradiated SUM159 and S68 cells to form colonies in a dose-dependent manner (**Figure 5G, H**). Of note, in MDA-MB-231 cells, the emergence of RTPs was not accompanied by an increase in YAP nuclear translocation, and LRP4 knockdown neither affected YAP pathway activation nor the ability of irradiated cells to form colonies. This suggests that the LRP4/YAP axis is not the sole mechanism underlying radio-resistance (**Supplementary Figure 5**). In this context, to further investigate the role of the LRP4/YAP axis in mediating the response to radiotherapy in additional models with more physiological relevance, we assessed the growth of patient-derived xenograft organoids (PDXO-CRCM389) following radiation therapy under wild-type (WT) or LRP4 knockdown (LPR4-KD) conditions. We observed a significant reduction of the growth capacity of PDXO silenced for LRP4 compared to the PDXO expressing LRP4 (**Figure 5I**). We also performed xenotransplantation assay of irradiated SUM159 cells LRP4-WT or LRP4-KD and observed a significant decrease of the capacity of LRP4-KD cells (shCTRL) to generate tumors compared to LRP4-WT cells (shLRP4) (**Figure 5J**).

Altogether, these results suggest that the LRP4/YAP axis is one of the key regulators of iCSC reprogramming and that LRP4 depletion may be a therapeutic opportunity to sensitize breast cancer cells to irradiation.

### LRP4 expression is increased in residual tumors post-chemotherapy and predict relapse-free survival in patients with breast cancers

**Figure 6:**
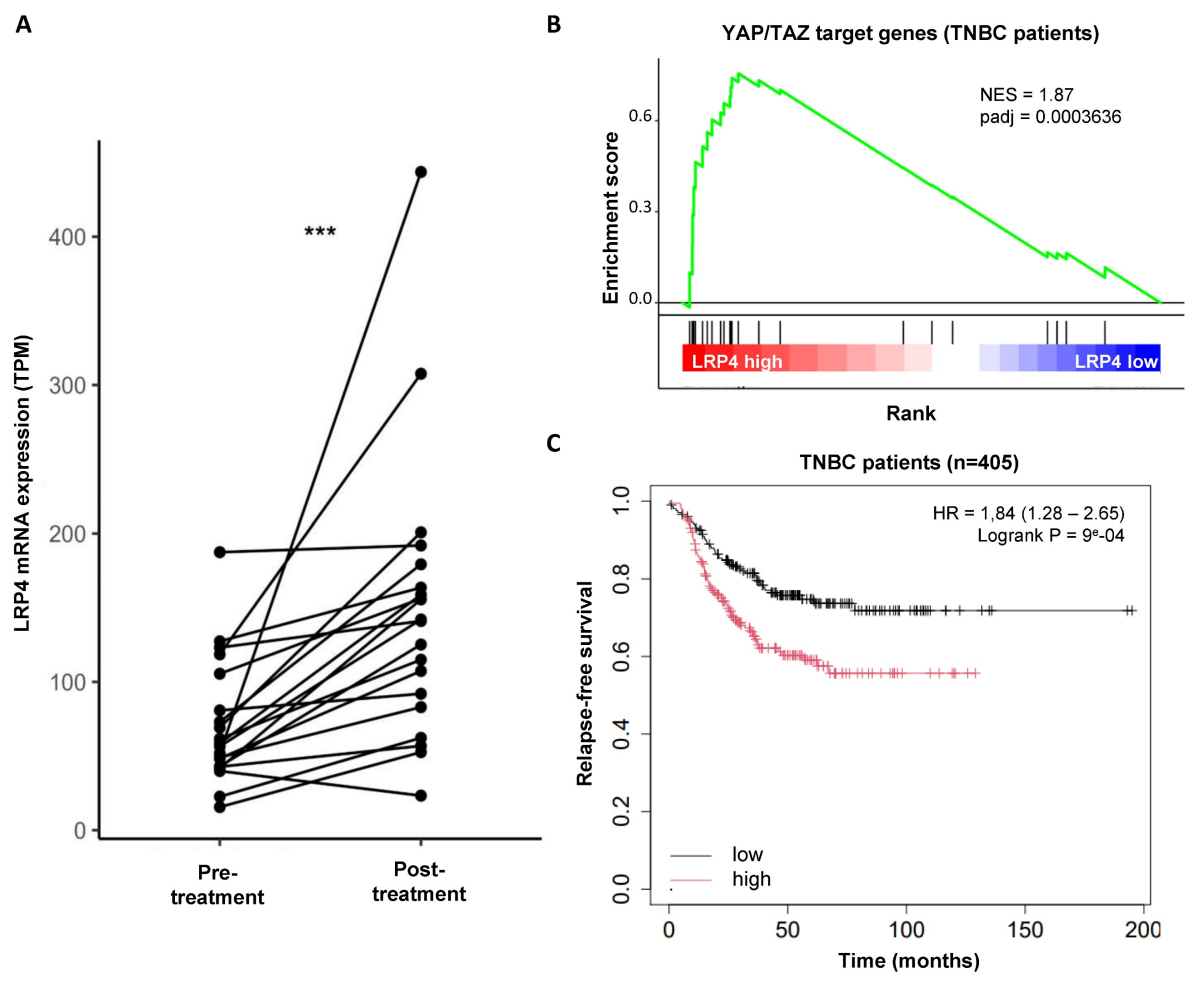
** Clinical association between LRP4 gene expression and breast cancer progression. A.** Interaction plot showing the effect of chemotherapy on LRP4 expression in patient with breast cancer following neo-adjuvant chemotherapy. Statistical test used is Wilcoxon paired test. ***p<0.001. **B**. Pre-ranked GSEA interrogating differential expression between LRP4 low and LRP4 high and YAP/TAZ target genes in triple-negative breast cancers (TNBCs). **C**. Kaplan-Meier relapse-free survival curve according to LRP4 expression levels in TNBCs. There is an association between a high level of LRP4 expression and poor prognosis (HR = 1,84 (1.28 - 2.65), Logrank P = 9^e-^04).

Having shown that LRP4 is essential for the emergence of iCSCs under radiation, we sought to investigate whether LRP4 expression is a more common biomarker of therapeutic response and could predict tumor recurrences. We used a dataset with gene expression of 20 paired breast cancer samples before and after chemotherapy (mainly Adriamycin/Cyclophosphamide, or capecitabine/docetaxel) 26. We observed that globally LRP4 expression increased in the remaining tumor suggesting a broader role for LRP4 in treatment resistance also including chemo- resistance (adjusted p value=1.5e-06) (**Figure 6A**). Interestingly, we performed a gene set enrichment analysis (GSEA) on RNA-seq data from 1992 breast tumors (METABRIC) and observed that tumors with a high expression of LRP4 were positively associated with YAP/TAZ target genes in all individual subtypes including TNBCs (**Figure 6B; Supplementary Figure 6**). This observation further confirmed that LRP4 expression is significantly associated with YAP/TAZ activation. To further evaluate the expression of LRP4 as a prognostic marker in patient treated by chemotherapy we used a series of 405 triplenegative breast cancers (TNBC) with matched gene expression and clinical data extracted from 7830 unique samples across 55 independent datasets 26. Patients with high level of LRP4 expression were associated with poorer relapse-free survival (RFS) than patient with a low level of LRP4 expression (HR=1.84(1.28-2.65), Logrank P = 9^e^-04) (**Figure 6C**).

Altogether, these observations suggest that LRP4 expression is a clinically relevant biomarker for predicting breast cancer progression. LRP4 appears as a key player in the generation of drug- as well as radiation-tolerant persister cells.

## Discussion

There is growing evidence that non-genetic processes are responsible for drug tolerance, which is a major obstacle to successful cancer treatment 4. Drug-tolerant persister (DTP) cells are becoming increasingly recognized as crucial contributors to the non-genetic process leading to adaptative resistance in a broad range of tumors in response to chemotherapy and targeted therapies 17,28-30. The emergence of DTP cells is thought to be the result of cellular reprogramming rather than the selection of naïve cells with an inherent ability to resist treatment. However, the capacity of a cancer cell to enter the DTP state remains a topic of debate. Some researchers propose an "equipotent" model, in which all cancer cells within a tumor have an equal potential to enter the DTP state 17, while others have identified only a small subset of cancer cells that are predisposed to enter the DTP state 31. Despite the wealth of evidence supporting the role of DTPs in fueling tumor recurrences, very little is known about the cellular mechanisms that underlie resistance to radiotherapy. Response to treatment, and in particular to irradiation, is the real challenge now facing breast cancer patients and the clinicians who care for them. Sensitizing the entire tumor to irradiation, in order to improve response and reduce tumor recurrence and metastasis, is a major avenue for improving breast cancer management. In our study, we utilized a lineage tracing system to demonstrate that radiation-tolerant persister cells originate from the non-cancer stem cell (CSC) subpopulation, rather than being selected from native CSCs. Thus, this observation parallels the emergence of DTP cells described in tumors after exposure to chemotherapy. Furthermore, we found that these radiation-tolerant persister cells possess stemness properties, which enable them to contribute to tumor recurrences.

If this non-genetic adaptation in cancer cells contributes substantially to the therapeutic evasion, it may also reveal new therapeutic opportunities. Two main approaches are currently explored with either blocking the capacity of cancer cells to enter treatment-tolerant persister state or to identify vulnerabilities of these residual treatment-tolerant cells. As proof of concept, it has been demonstrated that breast cancer cells exposed to taxanes enter an iCSC state through the activation of a Src family kinase-dependent pathway, and that inhibition of this pathway by dasatinib prevents cellular plasticity leading to iCSC generation, thus overcoming therapeutic resistance 5. More recently, it has been shown that DTP in colorectal cancers harbored a diapause-like state dependent on upregulation of the autophagy program and that targeting this pathway with an ULK1 inhibitor drastically reduced tumor recurrences 17. Therefore, both therapeutic approaches to either prevent or target DTP state are encouraging with a significant impact on tumor recurrence rate and need to be further explored.

In the context of radiation therapy, a recent study on colorectal cancer has shown that modifying the molecular machinery of persistent radiation-tolerant cells offers a new therapeutic strategy for improving response to radiation 32. Our study offers a molecular portrait of the radiation-tolerant persister cells that present a unique transcriptional identity. We did not identify a diapause-like state but rather an overexpression of DNA repair genes associated with an overexpression of WNT pathway genes.

Among the potential regulator of iCSC state, we identified the agrin co-receptor LRP4 and the subsequent downstream activation of YAP pathway. We demonstrated that LRP4 knockdown reduced nuclear YAP in iCSC, resulting in an increase of breast cancer radio-sensitivity. Recent studies identified LRP4 as a new player driving cell plasticity. The LDL receptor-related protein (LRP) family is an old family of proteins initially reported to regulate cholesterol homeostasis, but many data suggest they are implicated in a wide range of signaling pathways 33. LRP4 canonical pathway is activated during development and in neurological or neuro muscular diseases via Agrin-LRP4-MuSK signaling where it interacts with molecules such as the amyloid beta-protein precursor (APP) and WNT 34. More recently, the LRP4-MuSK pathway was shown to act as a mechanotransduction signal regulating YAP through the Hippo pathway 35,36. Because aberrant YAP/TAZ activity is known to drive cell plasticity to cause cell-fate switching 37, it may explain the importance of the LRP4/YAP axis in leading to the emergence of radio-induced CSC. In line with our observation, pancreatic ductal adenocarcinoma CSC appears to release extracellular vesicles (EVs) loaded in agrin that will bind to non-CSC via LRP4 to promote YAP activation and subsequently non-CSC reprogramming into CSC. Interestingly, PDAC patients with high levels of agrin and low inactive YAP show worse disease-free survival and treatment with anti-agrin significantly impairs tumor progression 23.

To our knowledge, this is the first time that LRP4/YAP axis has been implicated in breast cancer radiosensitization. Supporting our discovery, the modulation of Hippo pathway by blocking the KK-LC-1-FAT1 binding in TNBC has recently been shown to decrease ALDHbr CSC population and impairs tumor growth 38. Moreover, YAP/TAZ pharmacological inhibition appears to eliminate the chemo-resistant breast cancer stem cells 39. Such therapeutic opportunities may offer new approaches to limiting the emergence of radiation-tolerant persister cells and reducing tumor recurrences in breast cancer patients.

## Material & Methods

### Ethics statement

Animal studies were conducted in agreement with the French Guidelines for animal handling and approved by local ethics committee (Agreement no. #16487-2018082108541206 v3). Of note, mouse weight loss >20%, tumor necrosis, tumor volume >1500 mm3, ruffled coat + hunched back, weakness, and reduced motility were monitored daily and considered as endpoints.

### Animals

The NOD/SCID mouse colony was purchased from Charles River and grown in-house (CRCM animal core facility). Mice were maintained in individually-ventilated cages under specific pathogen-free conditions on a 12h light and 12h dark cycle and fed standard mouse chow ad libitum. Temperature was maintained between 20 and 24°C and the hygrometry between 40 and 60%. All experiments were performed under a hood with laminar flow. Mice were not subjected to any procedures prior to the xenotransplantation of human cells.

### Cell culture

SKBR7**,** MDA-MB-231, and MCF7 come from ATCC (https://www.atcc.org/). SUM159 was given by Dr. S.Ethier (Karmanos Cancer Center, Detroit, MI, USA), S68 was given by Dr. V. Castros (Université de Rennes, France) and BrCa-MZ-01 was given by Dr. R. Kreienberg (University of Ulm, Germany). All lines were grown in the standard medium as previously described 16*.*

### ALDEFLUOR assay

The analysis was processed on single-cell suspension from cell lines or PDXs. The ALDEFLUOR Kit (Stem Cell Technologies) was used to isolate the population with high aldehyde dehydrogenase enzymatic activity using an LSR2 cytometer (Becton Dickinson Biosciences) as previously described 40. To eliminate cells of mouse origin from the xenografts (CRCM404 or SUM159), we used staining with an anti-H2Kd antibody (#553563, BD Biosciences, 1:200, 20 min on ice) followed by staining with a secondary antibody labeled with phycoerythrin (PE; #115-116-146, Jackson Laboratories, 1:250, 20 min on ice).

### Irradiation

*In vitro*, cells grown as monolayers during 24 hours (2x10^4^ cells/well of 24-well plates or 5x10^5^ cells/T75cm^2^) and were irradiated at room temperature using a 6MV photon beam Elekta Synergy^®^ linear accelerator. Radiation treatment planning process was established by a medical physicist using the Pinnacle^®^ treatment planning system (Philips). The radiation dose delivered was ranging from 2 to 10 Gy according the experiment and with corresponding controls that were sham irradiated.

*In vivo*, to explore the efficiency of radiotherapy on tumor growth, we utilized a primary human breast cancer xenograft (e.g. CRCM404) generated from a chemo-naive triple-negative breast tumors 41. Cells from this PDX was transplanted orthotopically into fat pads of NSG mice. We injected 15,000 cells per fat pads of NSG mice (with one injected fat pad per mice) and monitored tumor growth. When tumor size was approximately 250 mm^3^, we initiated treatment. To limit eventual radiation therapy field borders and ensure adequate dose coverage to tumor area, a clip was positioned on the skin between the tumor and the abdomen and tumor was embedded in a piece of bolus (1.5 cm thick) that serve as a tissue equivalent material to enlarge the target volume. Then, using a CT scanner for 3D dosimetry (**Supplementary Figure 1**) we calculated optimal radiation planning process to administrated 10Gy to the tumor with limited dose to other normal structures. Mice were anaesthetized and maintain warm on a heat pad during all the radiation therapy procedure.

### Lineage tracing system

For each seven cell lines used in this study, cells were transduced with commercially available lentiviral particles (Vectalys) to engineer two derived cell lines expressing stably and constitutively either Tag-BFP (BFP^+^) or TurboRFP (RFP^+^) transgenes under control of EF1a promoter. Lentiviral infection was conducted by plating 2x10^5^ cells on 6-well plates and incubating them overnight (o/n) with 600 µL of culture medium, polybrene (8 μg/mL), and 1μL of lentivirus. Cells were then washed twice with PBS and expanded in their usual culture medium. Then, cell sorting was performed to enrich RFP^+^ and BFP^+^ cells.

To extemporaneously create a chimeric cell line, these two engineered cell lines were first labeled with ALDEFLUOR (as described earlier), and FACS-sorted (BD FACSAria™ III Cell Sorter). bCSCs (5% brightest cells in ALDEFLUOR channel) and non-bCSCs (10% dimmest cells in ALDEFLUOR channel) were isolated from the BFP^+^ and RFP^+^ cell lines, respectively, and mixed together to reconstitute a chimeric cell line by pooling 10% of BFP^+^/ALDH^br^ cells to 90% of RFP^+^/ALDH^-^ cells. These chimeric cell lines were expanded in their usual culture medium and the evolution of the expression of the lineage tracing system was assessed by FACS analysis.

Similar approach was developed *in vivo* to create a chimeric PDX-CRCM404. Following tumor dissociation, we first perform a magnetic cell separation to deplete mouse cells (Mouse depletion Kit, Miltenyi, 1/130 for 2x10^6^ cells) and then follow lentiviral transduction procedure previously described. Then, cells (BFP^+^ or RFP^+^) were xenotransplanted into mammary fata pads (1x10^5^ cells per fatpads) and resulting tumors harvested to proceed to the constitution of a chimeric PDX following the protocol described for cell lines. This chimeric PDX was re-implanted in new mammary fat pads to evaluate the cell state conversion with or without treatment.

### Tumorigenicity assay

SUM159 sorted-cell subpopulations (RFP^+^/ALDH^br^, RFP^+^/ALDH^-^, BFP^+^/ALDH^br^ BFP^+^/ALDH^-^) generated following irradiation of the chimeric cell line were xenotransplanted orthotopically into mammary fat pads of NSG mice. We performed serial dilution (with 5000, 500, 250, 100, 25, 10, and 1 cell per fat pad) to functionally evaluate the proportion of breast CSCs in each cell subpopulations. Each mouse that presents a tumor reaching a size of 25 mm3 was considered as a tumor bearing mouse. The frequency of breast cancer stem cells was determined using the Extreme Limiting Dilution Analysis. Cells isolated from tumors generated by ALDH^br^/BFP^+^ cell subpopulation were sorted according to their ALDH activity and reimplanted into secondary mice, in serial dilution. To evaluate the impact of LRP4 knockdown on tumorigenicity potential, we first transfected SUM159 cells with lentivirus vector expressing shRNA constructs (shLRP4, #SHCLNV or shCTRL, #SHC201VN (empty vector); Merck Sigma-Aldrich). Lentiviral infection was conducted by plating 250,000 cells on 6-well plates and incubating them overnight with 1mL of culture medium, polybrene (8 μg/mL), and 5-10 μL of lentivirus (MOI = 2). Cells were then washed twice with PBS and expanded during 10 days in their usual culture medium supplemented with puromycin (2μg/ml) for selection. Puromycin resistant cells were then re-plated (5x10^5^ cells/T75cm^2^) and irradiated (10Gy) and xenotransplanted 24 hours later into mammary fat pads of NSG mice (100K-200k cells per fat pad). Each mouse that presents a tumor reaching a size of 50 mm3 was considered as a tumor bearing mouse.

### Tumorsphere assay

SUM159 sorted-cell subpopulations (RFP^+^/ALDH^br^, RFP^+^/ALDH^-^, BFP^+^/ALDH^br^, BFP^+^/ALDH^-^) generated following irradiation of the chimeric cell line were plated in single-cell suspension in 96-wells ultra-low attachment plates, in a serum-free mammary epithelium basal medium 39. The frequency of cancer cells with tumorsphere-forming potential was determined using the Extreme Limiting Dilution Analysis by plating cells at 25/10/5/3/2 and 1 cell per well (n = 18-36 wells/conditions). The number of wells containing at least one sphere after 10 days of culture was considered as positive.

### RNA extraction

Total RNA was isolated using Maxwell RSC simply RNA Tissue Kit according to manufacturer's instructions.

### RNA-seq

Total RNA was extracted as described above and its quality was assessed by Tapestation (only samples with RIN score > 8 were considered for sequencing). RNA-Seq libraries were prepared using the Swift RNA Library Kit (Swift Biosciences, Cat#R1024 and R1096) according to manufacturer's instructions and sequenced on an Illumina NovaSeq 6000 (PE100). Both sets of libraries were sequenced at the MGX Next Generation Sequencing Core Facility (IGH, Montpellier). Differential expression analysis comparing each cell subpopulation was performed with DESeq2 42. For each cell compartment, all expressed genes were pre-ranked according to their fold-change and adjusted p-value. Cluster's signatures were computed as the mean of genes belonging to the signature on the scaled and centered matrix. The incidence of the four cell state identities for each of the three cluster's signature was assessed by computing a metagene-based score defined for each sample by the mean of cluster associated genes expression. Gene set enrichment analysis (GSEA) (http://www.broadinstitute.org/ gsea/) was used to identify a priori defined sets of genes that were differentially expressed between iCSC and late non-CSC or iCSC and CSC. For each cell subpopulation, significant genes were filtered with a log2(fold-change) threshold of 1 and a p-value threshold of 0.05, and gene ontology analysis was performed using the MSigDB database and clusterProfiler 42. Collections C6 (oncogenic signatures), H (hallmark gene sets), C2 (curated gene sets) and C5 (GO gene sets) were screened. Concerning enrichment of the YAP/TAZ target genes (22-gene signature 25, we performed a GSEA on pre-ranked differentially expressed genes between iCSC and late non-CSC.

### RNAi screening

An automated screening routine was developed on a robotic workstation equipped with a 96-well head probe (Nimbus, Hamilton) to screen a small siRNA library (80 target genes, 1 siRNA pool per gene, On-Target Plus pooled siRNAs, Horizon Discoveries). Briefly, siRNA pools were lipoplexed with Lipofectamine RNAiMAX (Life Technologies) in collagen-coated, clear bottom, black-walled 96-well culture plates (Costar, Cat# 3904). After 15 min of complexation, the chimeric SUM159 cell line was seeded on top of the lipoplexes (3 000 cells/well; final [siRNA] = 20 nM) and incubated for 3 days at 37°C and 5% CO2 in a humidified incubator. Each pooled siRNA from the library was transfected as six separate replicates in six independent culture plates. Each culture plate also received various positive and negative controls: ten wells received the transfection reagent alone (“MOCK” well, negative controls), ten were transfected with a pool of four scrambled siRNAs (“NEG” Wells, negative control, ON-TARGETplus Non-targeting Pool, Dharmacon), and two were transfected with an siRNA targeting KIF11 (“EG5” wells, positive control, custom siRNA, target sequence: AACTGAAGACCTGAAGACAAT, Qiagen). Additionally, two wells were left untreated to receive the DEAB control during the ALDEFLUOR assay. Plates were then incubated at 37°C and 5% CO2 in a humidified incubator. 24h post-transfection, well volumes were completed to 200µL with complete medium, plates were irradiated with 6 MV X-rays (single fraction of 10 Gy, delivered in less than 3 minutes, Versa HD, Elekta), then immediately returned in the incubator.

Three days post-irradiation, the cell amount and the %ALDH^br^ cell amount (=%CSC) in the SUM159 RFP^+^ and the SUM159 BFP^+^ subpopulations were assessed using a previously described adaptation of ALDEFLUOR assay (Stem Cell technologies) for cell imaging and analysis in microplate format 18. Nine fields per well were acquired at 10× magnification, in four fluorescence channels: green for ALDEFLUOR (ex: 470 ± 10 nm; em: 525 ± 25 nm), blue for Tag-BFP (ex: 380 ± 20 nm; em: 445 ± 35 nm), red for Turbo-RFP (ex: 535 ± 15 nm; em: 595 ± 35 nm), and far red for DRAQ5 (ex: 630 ± 20 nm; em: 705 ± 55 nm).

An automated algorithm was developed under Harmony 3.0 (Perkin Elmer) to quantify the Total cell amount and the %CSC in the SUM159 RFP^+^ and the SUM159 BFP^+^ subpopulations. Briefly, nuclear regions of interest (ROI), segmented in the DRAQ5 channel, were used to quantify the Total cell amount. Cells were labelled as SUM159 BFP^+^ or SUM159 RFP^+^ according to their blue and red average fluorescence in the nuclear ROI, respectively. Cells were defined as ALDH^br^ when their average background-corrected ALDEFLUOR signal in the nuclear ROI was found above the one measured in the DEAB condition. %CSC was computed as the amount of ALDH^br^ cells in each subpopulation over the total cell amount in the corresponding subpopulation. Each candidate gene identified by the siRNA screening were validated by flow cytometry using the ALDEFLUOR assay on chimeric SUM159 cell line after radiotherapy treatment.

### qRT-PCR

cDNA was synthesized from 1 mg of RNA with the Transcriptase inverse SuperScriptII kit. Real-time PCR amplification and analysis were conducted with the TaqMan Universal Master Mix II with UNG on a 7500 Real-Time PCR System (Applied Biosystems). RNA levels were normalized to ACTB expression using the DDCt method. Probe; YAP1 (Hs00902712_g1), AXL (Hs01064444_m1), NT5E (Hs00159686_m1), CYR61 (Hs00155479_m1), ARHGEF17 (Hs00998246_m1), ASAP1 (Hs00987469_m1), RBMS3 (Hs01104892_m1), GADD45A (Hs00169255_m1), AMOTL2 (Hs01048101_m1), CCDC80 (Hs00277341_m1), MYOF (Hs00203853_m1), FOXF2 (Hs00230963_m1), PTPN14 (Hs00193643_m1), CRIM1 (Hs01070663_m1), LATS2 (Hs01059009_m1), DOCK5 (Hs00227848_m1), CTGF (Hs00170014_m1), ANKRD1 (Hs00173317_m1), NUAK2 (Hs01011402_m1), IGFBP3 (Hs00181211_m1), TGFB2 (Hs00234244_m1), FJX1 (Hs00534909_s1), F3 (Hs01076029_m1).

### Colony-assay

To evaluate the effect of LRP4 silencing on radiotherapy-response, SUM159, S68, and MDA-MB-231 cells were first transduced with a lentivirus expressing shRNA constructs (shLRP4, #SHCLNV or shCTRL, #SHC201VN (empty vector); Merck Sigma-Aldrich)..Cells shCTRL or shLRP4 were plated at low-density (between 75 and 2000 cells per well, depending on the dose) in 12-well plates (Falcon, 353043), with 3.5mL of culture medium and irradiated between 0 and 6Gy using Synergy linear accelerator (Elekta). After 7-8 days, cells were washed with PBS (Gibco, 14190-094), and stained with a 1:1 solution Ethanol (Carlo Erba, 4146322) - Crystal Violet (Merck, 61135, resuspended in H_2_O at 0.5%) for 10 minutes at room temperature. After staining, cells were washed two times with H_2_O. Colonies were manually counted. Survival for a given well was computed as following:

Survival = (number of colonies in treated well / number of cells plated by treated well) / (mean number of colonies in untreated wells / number of cells plated by untreated well)

### Immunofluorescence

After cell sorting, cells were cytospun and fixed with 4% paraformaldehyde for 10' and permeabilized with 0.1% Triton X-100 for 5' before blocking with protein block (Dako). Cells were labeled 1 hour at room temperature with anti-YAP (Sc-101199, clone 63.7, Santa Cruz, 1/200). After 10' of wash with TBST, cells were incubated for 30' with anti-mouse (A-11029, ThermoFisher), 1/500). DNA was counterstained with DAPI 4′,6-diamidino-2-phenylindole (Invitrogen, ProLong Gold antifade reagent with DAPI, P36935). Images were acquired using Nikon AX confocal microscope equipped with a 63× objective. For each condition, immunofluorescence scoring was done on 100 cells in three independent experiments.

### Immunoblot analysis

Cells were lysed in ice-cold lysis buffer containing Hepes 50 nM, pH7.5, EDTA 1mM, pH 7, NaCl 150 mM, NaF 100mM, Na3VO4 1mM, Triton X-100 1%, and complete Proteinase Inhibitor Cocktail (Roche, #04693159001). Cell lysates were migrated in 4-12% SDS-PAGE (Sodium Dodecyl Sulfate-PolyAcrylamide Gel Electrophoresis). The following primary antibodies were used: anti-YAP/TAZ (rabbit mAb, #8418, Cell Signaling, 1/1000), anti- p-YAP (ser39) (Rabbit mAb, Cell Signaling, #13619, 1/1000). Detection of GAPDH (Rabbit pAb, Cell Signaling, 1/5000) was used as loading control.

### Patient-derived xenograft organoids (PDXO)

To grow organoids from PDX model (CRCM389), 250,000 cells were resuspended in 28µL of culturex (Biotechne), seeded on a 48-well plate, and cultured in 400µL of medium supplemented with 10µM of L-Y27632 (Sigma, G9145) as previously described 44. After 5 culture days, PDXOs were dissociated with TrypLE Express 1X (Gibco, #12605-010) during 15 minutes at 37°C under agitation (155 RPM) to obtain a single-cell suspension. To perform the silencing of LRP4 in PDXO, we used lentivirus expressing shRNA constructs (shLRP4, #SHCLNV or shCTRL, #SHC201VN (empty vector); Merck Sigma-Aldrich). PDXO transfection was performed using previously described protocol 45. Briefly, viral particles were added at a MOI of 2 to RetroNectin-coated plates (40 mg/mL), which were then centrifuged at 1,000xg for 1 h and incubated at 37°C for 3 h. PDXO single-cells suspension were added to the plates at 1x10^6^ cells/mL in 500 mL of PDXO medium in the presence of protamine sulfate (20 mg/mL). To increase transduction efficiency, plates were centrifuged at 1,000xg for 1 h and incubated at 37°C overnight. The next day, fresh PDXO medium was added to each well and PDXO cells were irradiated at room temperature using a 6MV photon beam Elekta Synergy^®^ linear accelerator. We delivered a dose of 8Gy in the treated condition versus no treatment. Pictures were taken after 7 days of culture and compared to PDXO's picture before treatment (EVOS microscope, Thermo Fisher Scientific). PDXOs size were evaluated in inch^2^ using the particles analysis tool from ImageJ.

### Relapse-free survival in public primary tumor datasets

To evaluate the impact of LRP4 expression on breast tumor relapse-free survival (RFS), we used the online survival analysis platform “Kaplan-Meier Plotter” (https://kmplot.com/) 27. Clinicopathological and mRNA expression data of breast cancer samples from 55 public data sets were collected from Gene Expression Omnibus (GEO, NCBI). Data analysis was done on a subset of 405 patients with triple-negative breast cancers (TNBCs). Survival was calculated using the Kaplan-Meier method, and curves were compared with the log-rank test.

### LRP4 expression in tumor biopsies

To evaluate LRP4 gene expression evolution before and after chemotherapy, we interrogated RNA-sequencing profiles of 22 matched pre- and post-treatment breast tumors 25. Differential expression analysis comparing paired tumor samples was performed with DESeq2 42. To define the association between LRP4 expression and YAP/TAZ target gene activation, we used the METABRIC dataset 46, a public dataset of human breast cancer containing 1992 tumors, with tumor subtypes classified according to the PAM50 classification. Each tumor was characterized based on LRP4 expression. We considered the first quantile as tumors with low LRP4 expression, while the last quantile represented those with high LRP4 expression. Differential expression analysis comparing tumors with low and high LRP4 expression (996 tumors) was performed separately within each tumor subtype (208 triple-negative breast cancers, 350 luminal A tumors, 238 luminal B tumors and 112 Her2 tumors) using limma 47. All expressed genes were pre-ranked according to their fold-change. Pre-ranked gene list was tested by GSEA for its enrichment on the YAP/TAZ target genes (22-gene signature 42).

### Statistical analysis

Graphpad Prism 5.0 was used for data analysis. The results are presented as mean ± SD for at least three repeated independent experiments. To investigate associations among variables, univariate analyses were performed using nonparametric nonparametric Wilcoxon rank-sum test, chi-squared test or Fisher's exact test when appropriate. Statistical analysis considered unequal variance and applied the Welsh degrees-of-freedom correction when using parametric analysis. Extreme limiting-dilution analysis (http://bioinf.wehi.edu.au/software/elda/) was used to evaluate breast CSC frequency. In all cases, a p-value < 0.05 was considered as statistically significant. To compare the LRP4 expression in paired tumor samples, the calculation of adjusted p-value was performed with paired analysis using DESeq2 (v1.38.3).

## Supplementary Material

Supplementary figures.
