# Supplementary material for: The LRP4/YAP axis drives the radiation-tolerant persister (RTP) cell state in breast cancer
Source: Theranostics. 2025 Jun 23;15(15):7528–44. doi: 10.7150/thno.101393 (PMC12325171; doi:10.7150/thno.101393)
Supplement: Supplementary file 1 — Supplementary figures. [file thnov15p7528s1.pdf]

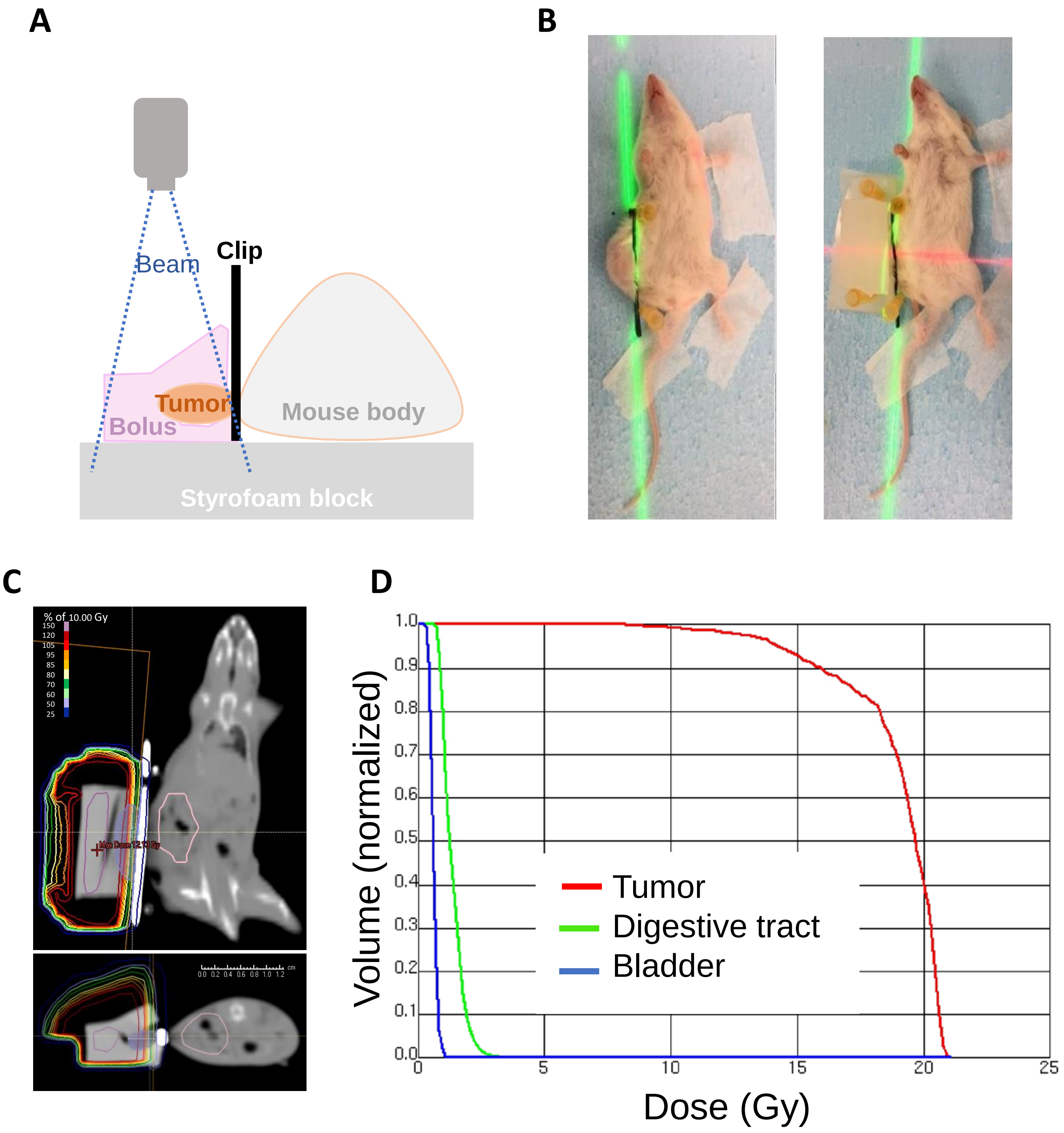

Supplementary Figure 1

Supplementary figure legend

Supplementary Figure 1. Protocol of radiation therapy on patient-derived xenograft (PDX). A-B. Schematic representation of the experimental protocol for PDX irradiation (**A**), with representative photos without (left panel) and with bolus (right panel) (**B**)**. C-D**. Radiation dose optimization procedure for PDX irradiation with a representative CT scan (**C**) and the associated dose-volume histograms (DVHs) of the tumor (in red) and critical organs (digestive tract in green, and bladder in blue) (**D**).

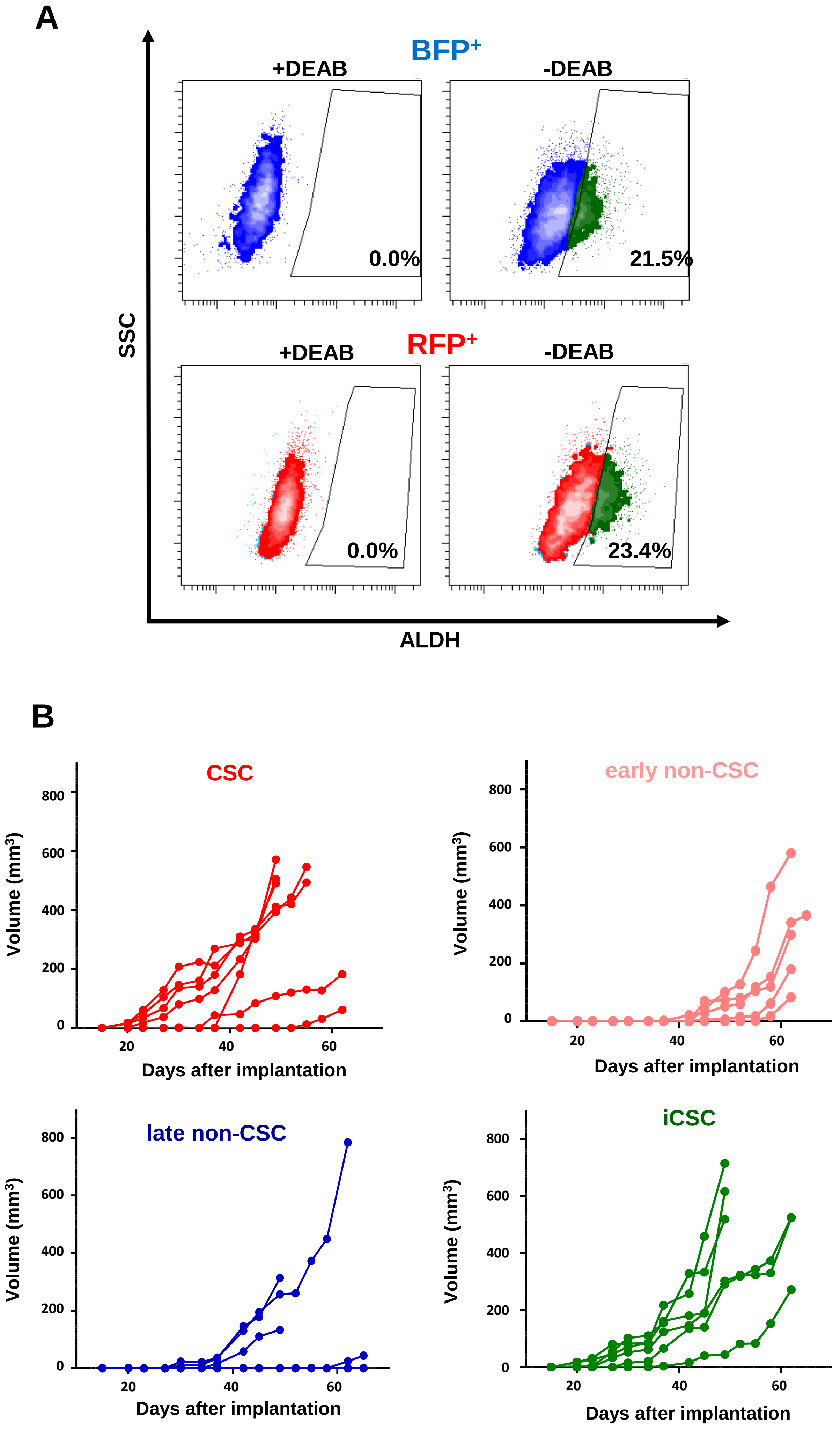

Supplementary Figure 2

Supplementary Figure 2. Tumor growth kinetic of each SUM159 cell subpopulations post-irradiation. A. Representative examples of FACS plot for ALDH activity in SUM159 engineered to express BFP (blue fluorescent protein) or RFP (red fluorescent protein). DEAB is an ALDH inhibitor used as negative control. **B**. Each panel represents the growth kinetics of one SUM159 cell subpopulation (CSC, early non-CSC, late non-CSC, iCSC) from one individual injection of 5,000 cells (n=5 per subpopulation).

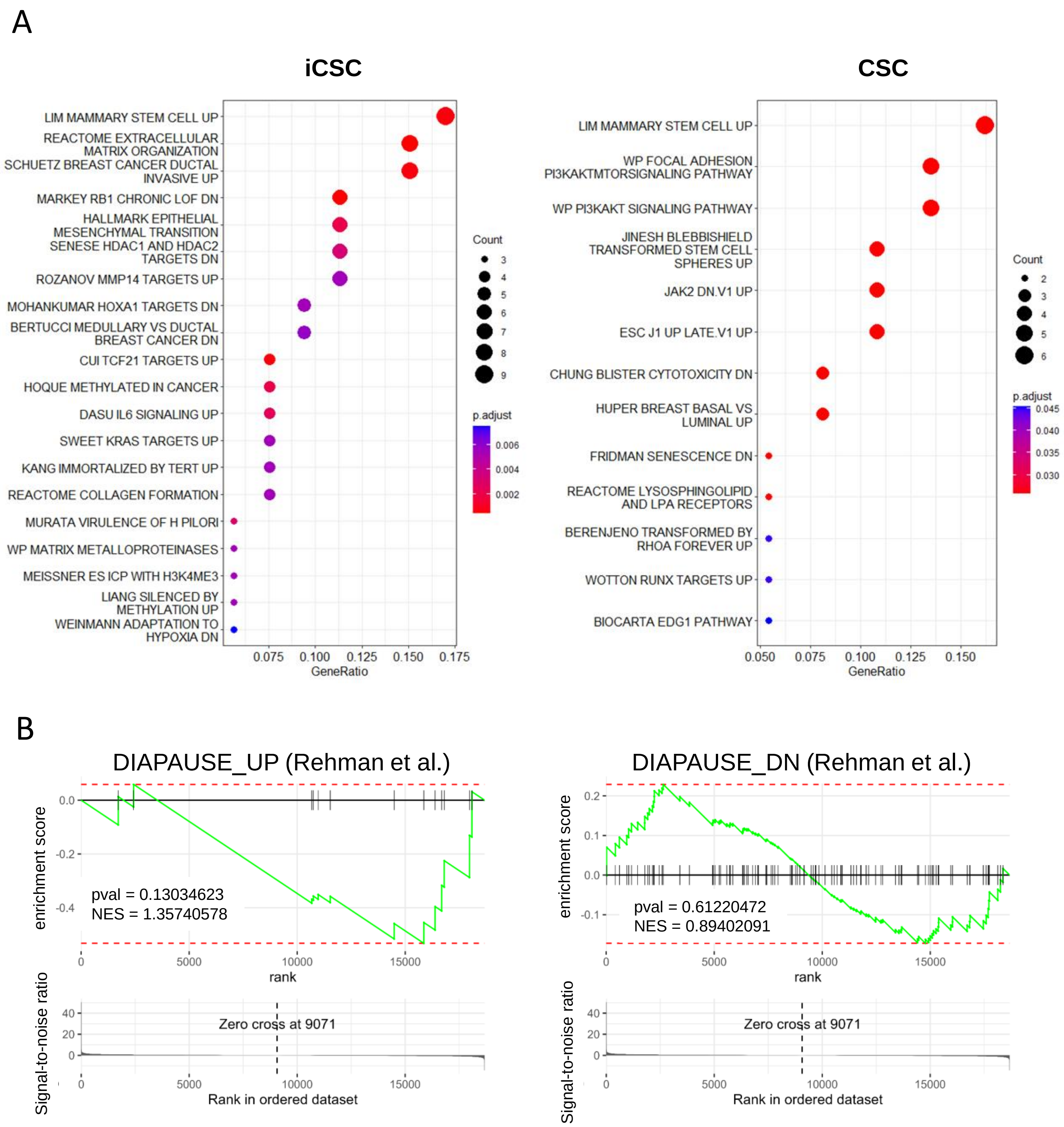

Supplementary Figure 3

Supplementary Figure 3. The radiation-tolerant persister cells gene expression profile. A. Bubble graph for GSEA-based MSigDB collections analysis revealed enriched pathways in iCSC compared to CSC. **B.** GSEA analysis comparing the iCSC versus the late non-CSC signature, to the ranked transcriptome of the diapause signature detected in DTP colorectal cancer cells (upregulated genes, UP; downregulated genes, DN).

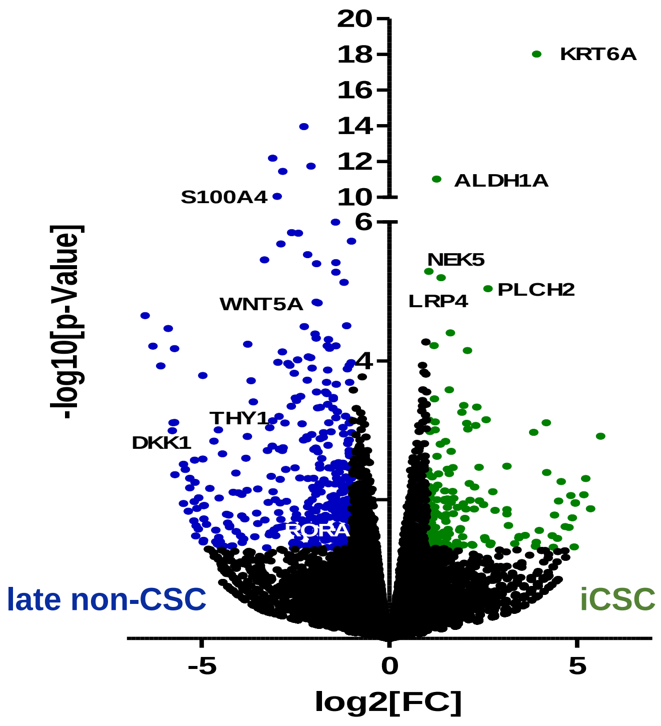

Supplementary Figure 4

Supplementary Figure 4. The radiation-tolerant persister cells gene expression signature. Volcano plot of the distribution of differentially expressed genes (p-value adjusted < 0.01) in iCSC, relative to late non-CSC. Genes of particular interest are indicated.

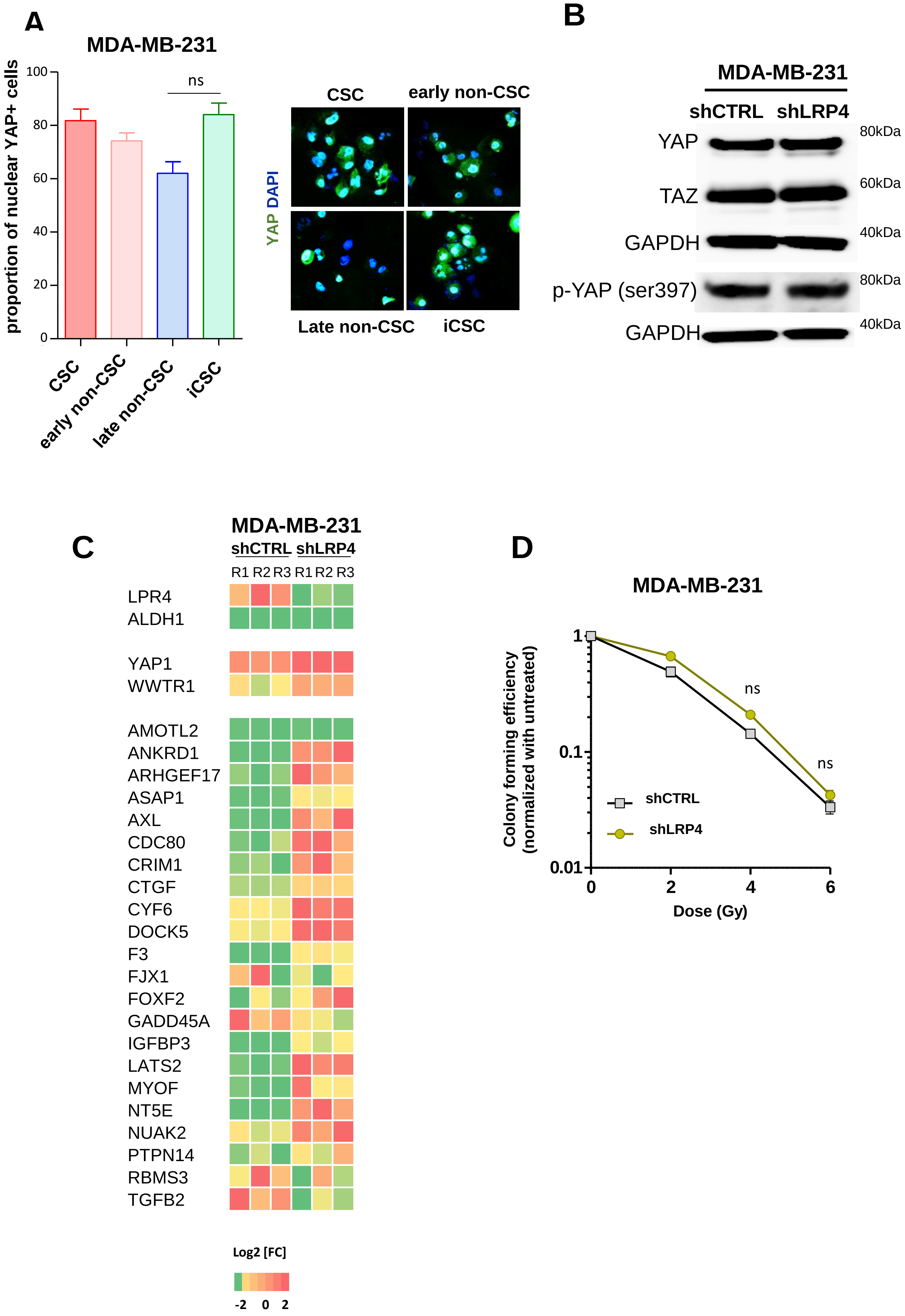

Supplementary Figure 5

Supplementary Figure 5. LRP4/YAP axis inhibition did not radiosensitize MDA-MB-231 iCSC. A. Proportion of MDA-MB-231 cells presenting a nuclear location of YAP in each cell subpopulations five days post-irradiation, with representative images of YAP staining (in green) in each cell subpopulations on the right panel. Nuclei are counterstained with DAPi (in blue). **B**. Western blot of markers related to YAP/TAZ signaling and its activation in MDA-MB-231 cells silenced for LRP4 (shLRP4) compared to the non-targeting shRNA (shCTRL). The mean intensities are indicated below each band for each condition. **C**. Heat map representing the mRNA expression of the LRP4, ALDH1A1, and YAP/TAZ target genes in MDA-MB-231 silenced for LRP4 (shLRP4) compared to the non-targeting siRNA (siCTRL). Each row represents three independent replicates per conditions (R1, R2, and R3). **D**. MDA-MB-231 cells following LRP4 silencing (shLRP4) compared to a non-targeting siRNA (shCTRL), were exposed to various dose of radiation therapy and subjected to clonogenic survival assays, with representative images (right panels). Statistical test used is Student's t-test. Data represent mean ± SD. ns (not significant).

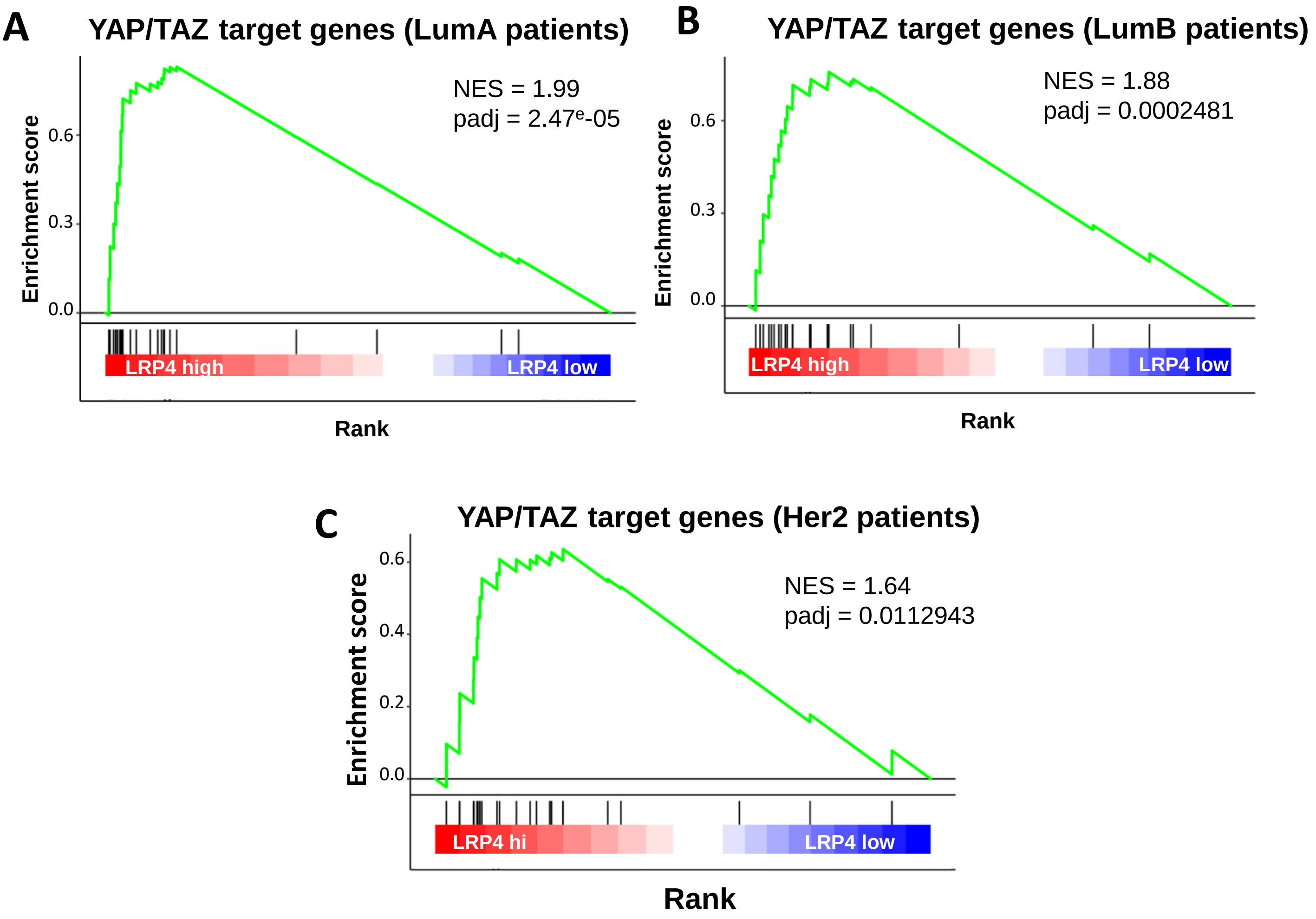

Supplementary Figure 6

Supplementary Figure 6. Gene expression association between LRP4 and YAP/TAZ target genes in Luminal A, Luminal B, and Her2 patients. A-C. Pre-ranked GSEA interrogating differential expression between LRP4 low and LRP4 high Luminal A (**A**), Luminal B (**B**), and Her2 tumors (**C**) of YAP/TAZ target genes.
